# Optimization of grape juice deacidification using mixture of adsorbents: A case study of Pekmez

**DOI:** 10.1002/fsn3.1586

**Published:** 2020-04-21

**Authors:** Mohammad Rezaei, Mohammad Alizadeh Khaledabad, Ehsan Moghaddas Kia, Zahra Ghasempour

**Affiliations:** ^1^ Department of Food Science and Technology Afagh Higher Education Institute Urmia Iran; ^2^ Department of Food Science and Technology Urmia University Urmia Iran; ^3^ Department of Food Science and Nutrition Maragheh University of Medical Sciences Maragheh Iran; ^4^ Department of Food Science and Technology, Nutrition and Food Sciences Faculty Tabriz University of Medical Sciences Tabriz Iran

**Keywords:** adsorbent, calcium carbonate, grape syrup, neutralization, tartaric acid

## Abstract

Grape syrup (Pekmez or Dooshab) is one of the nutritious products developed through grape processing. One of the main challenges in the industrial manufacture of this product is the utilization of traditional *pekmez* earth for tartaric acid adsorption. The objectives of this study were to investigate the effect of calcium carbonate, nano‐silica, alumina, and activated carbon as adsorbents and also contact time in grape juice deacidification, and to determine the effects of these adsorbents on the physicochemical properties of grape juice by using the Box–Behnken statistical design. By applying different amounts of these adsorbents in grape juice, the magnitude of acidity decrement and the physicochemical properties such as acidity, pH, transmittance, the amount of reducing sugars, formalin index, and adsorption efficiency were investigated. Data analysis showed that different mixtures of adsorbents at different concentrations had significant effects on acidity and pH of the samples but no effects on the level of reducing sugars and formalin index were observed (*p* > .05). According to the results, the adsorption capacity with the highest calcium carbonate content (0.7 g/100 ml) was about 88%; the maximum acidity decrements of up to 92% were achieved using the treatments containing calcium carbonate, nano‐silica, and activated carbon, while alumina failed to affect the acidity of the samples. Optimum conditions were obtained in 1.27, 0.21, 0.7, and 0.07 g/100 ml alumina, nano‐silica, calcium carbonate, and activated carbon, respectively, resulting pH 4.3 and acidity 0.37% in grape syrup.

## Graphical abstract

Grape syrup (reduced acid product) is one of the nutritious products developed through grape processing. The objectives of this study were to investigate the efficiency of using mixtures of adsorbents in the separation of tartaric acid, and to determine optimum adsorbents for acidity decrement beside optimum physicochemical properties.

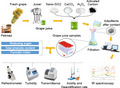

## 1 INTRODUCTION

Grape syrup contains large amounts of natural sugars including glucose, fructose, and sucrose and minerals such as calcium and iron, so that it can be used to treat anemia, in addition to providing a good source of vitamins (A, C, B2, and B1), organic acids, and some antioxidant agents such as phenolic and flavonoids. Due to the high levels of monosaccharides (glucose and fructose), grape syrup can be digested and adsorbed to support immediate energy; its average energy value is 293 kcal/100 g. Therefore, it is valuable in situations demanding urgent energy, and it could play an important role in the diet of different age groups, especially children and athletes (Akbulut & Ozcan, 2009; Heshmati, Ghadimi, Ranjbar, & Khaneghah, 2019; Heshmati, Ghadimi, Ranjbar, & Mousavi Khaneghah, 2020). Grapes and grape products contain a wide range of polyphenolic constituents that have been reported to show anticancer and anti‐inflammatory effects in vitro, as well as the ability to block cellular events that lead to atherosclerosis and coronary heart disease. The compounds presumed to provide these positive health effects are mainly flavonols, procyanidins, anthocyanins, and phenolic acids (Capanoglu, de Vos, Hall, Boyacioglu, & Beekwilder, 2013). Grape syrup, as one of the most popular traditional food products in eastern culture (Heshmati et al., 2019), is a concentrated and shelf life‐extended form of grape juice formed by boiling without the addition of sugar or other food additives after the acids are removed (Kaya & Belibagli, 2002).

**SCHEME 1:**
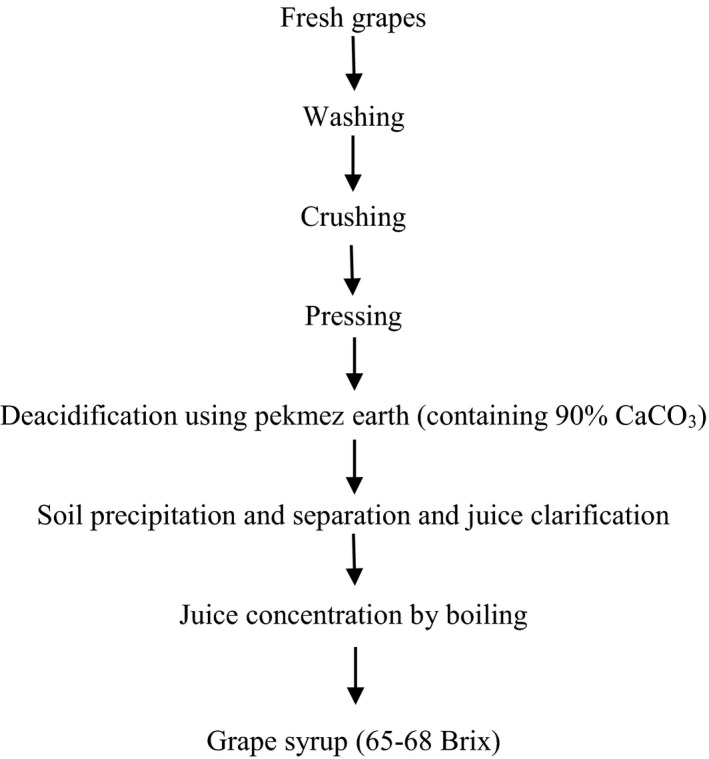
Production diagram of grape syrup from fresh grapes (Kaya & Belibagli, 2002).

Flow diagram of a typical grape syrup processing operation is presented in the Scheme 1. During the processing of grape syrup, a calcareous soil called “grape syrup earth,” containing approximately 90% calcium carbonate is added to grape juice. The grape syrup earth lowers the acidity caused by naturally existing tartaric and malic acids by precipitating them as calcium tartrate and calcium malate. Grape juice is concentrated usually in open vessels and rarely under vacuum to obtain 65–68° Brix; this product is called liquid grape syrup (Kaya & Belibagli, 2002). It is also known by the names of “Pekmez” and “Petimezi” in Turkey and Greece, respectively. The most important problem in grape syrup production is grape syrup earth addition, as an acid adsorbent, into the juice. Due to the presence of heavy metals in soil composition containing different calcium purities, this can be very harmful to the human body and cause kidney or liver disease, etc (Jarup, 2003). Also, soil usage in grape syrup production causes other problems, such as high consumption of soil, high waste production, high costs, limitation of daily production, and low performance of the production line. In recent years, the usage of nanoparticles and chemical adsorbents has been studied for the adsorption studies.

Calcium carbonate (CaCO_3_) with a pH of 9 is a highly adsorbent material used in various industries, especially for the neutralization of juices. It combines with tartaric acid and malic acid to form calcium tartrate crystals and calcium malate (Kaiser, 2012). Silica (SiO_2_) has adsorbent hydroxyl groups and a large atomic structure. It is one of the most important adsorbents for the separation of materials in chemical technologies and chromatography. Factors influential on the ability of silica to adsorb include: pH, temperature, and ionic strength; silica is apparently more efficient for the adsorption of cations relative to anions (Papirer, 2000). Alumina (Al_2_O_3_) is highly resistant to various chemical environments and can withstand extreme stress and maintain its properties until up to 1,500°C (Uslu & Inci, 2009). The utilization of activated carbon is a widely used technique for reducing organic pollutants from water and is most used in the food industry for bleaching and removing heavy metals or phenolic compounds (Farouk Mohamed, 2011).

In recent years, many similar researches about tartaric acid adsorption have been done. A study by Kontogiannopoulos, Patsios, and Karabelas (2016) showed that tartaric acid recovery from winery lees using Lewatit^®^ MonoPlus S108 as a strongly acidic cation exchange resin at room temperature is possible. In that study, the pH, water dosage, and cation exchange resin dosage were the three variables that were investigated (Kontogiannopoulos et al., 2016). Another study was conducted by Shayanfar and Bodbodak (2014) on the effect of different physicochemical detartration methods on red grape juice quality. In that study, the application of cold treatment, tartar cream (potassium bitartrate), carboxymethyl cellulose (CMC), and mannoprotein were investigated. Then, all samples were stored at 5°C to stimulate tartrate crystal precipitation and were investigated for changes in color indices, sediment levels, acidity, pH, and taste. Among these methods, the addition of mannoprotein and CMC proved to have the ability to stabilize tartrate crystals in red grape juice (Shayanfar & Bodbodak, 2014). The adsorption equilibria of L‐ (+)‐tartaric acid onto alumina from wastewaters of wineries were studied by Uslu and Inci (2009). In that study, the adsorption experiments were carried out at three different temperatures (298, 310, and 325 K). The adsorption of L‐ (+)‐ tartaric acid was found to be dependent on the acid concentration and the amount of alumina. The maximum percentage removal of L‐(+)‐tartaric acid was 22% using alumina at 298 K.

The aim of this research was to find the best mixture of nano‐silica, calcium carbonate, alumina, and activated carbon as acid adsorbents for grape syrup production. In this research, the pH, titratable acidity and deacidification rate, transmittance, turbidity, Brix, reducing sugars content, polyphenol content, and formalin index of grape juice samples introduced with mixtures of adsorbents were investigated and the proper mixture of adsorbents was obtained through statistical optimization.

## 2 MATERIALS AND METHODS

### 2.1 Materials

Grapes (*Vitis vinifera* “Trebbiano”) were obtained from a local grape farm (Urmia, Iran) as the raw materials. Nano‐silica was prepared by Pioneer Iranian Nano Materials Company. Calcium carbonate, alumina, activated carbon, and other laboratory compounds and solutions were obtained from Merck.

### 2.2 Statistical design and analysis

The present study was conducted in two steps, through two experimental designs. At the first stage, a mixture experimental design was applied to investigate the effect of four components (nano‐silica: 0%–20%; calcium carbonate: 0%–100%; alumina: 0%–100%; and activated carbon: 0%–10%), with total amount of 0.7 g/100 ml, on physicochemical properties of grape juice which was carried out across 24 treatments in four blocks. According to the results of the first step, the effects of two variables, namely calcium carbonate and activated carbon, were highly significant. Due to the high uptake of the acid by these two adsorbents and thus their great impact, the second step was performed using a Box–Behnken experimental design to investigate the effect of three factors, nano‐silica amount, alumina amount, and contact time (Table 1). The calcium carbonate and activated carbon amount were constant in all treatments (0.7 and 0.07 g/100 ml, respectively). The number of treatments was 16; these treatments were tested, and the data analyzed and optimized against a control sample (grape juice). Analysis of variance (ANOVA) at 95% confidence level (*p* < .05) was performed, and Design‐Expert 7 software was used for analyzing data, optimizing, and plotting curves. Data were fitted to second‐order polynomial Equation 1 for each dependent *Y* variable, through a stepwise multiple regression analysis.

(1) $$Y=\beta_{0}+\sum \beta_{i}x_{i}+\sum \beta_{\mathrm{ii}}x_{i}^{2}+\sum \beta_{\mathrm{ij}}x_{i}x_{j}$$

where *Y* = predicted response, *β*_0_ = a constant, *β_i_* = linear coefficient, *β_ii_* = squared coefficient, and *β_ij_* = interaction coefficient.

**TABLE 1:** Box–Behnken experimental design for three numeric variables: alumina, nano‐silica, and time of contact

Run	A: alumina (g/100 ml)	B: nano‐silica (g/100 ml)	C: time of contact (min)
1	0	0.4	135
2	1.5	0.2	240
3	1.5	0	135
4	0.75	0.2	135
5	0	0.2	30
6	1.5	0.2	30
7	0.75	0.2	135
8	0.75	0.2	135
9	0.75	0.4	240
10	0.75	0	30
11	0.75	0.4	30
12	0.75	0	240
13	0.75	0.2	135
14	1.5	0.4	135
15	0	0	135
16	0	0.2	240

### 2.3 Preparation of grape juice

Firstly, all grapes were washed and clustered. The grapes were then pressed with a juicer and filtered. The volume of each sample was 400 ml to begin the test. In the next step, calcium carbonate, nano‐silica, alumina, and activated carbon adsorbents were added into the samples according to the experimental design (Table 1). The amounts of calcium carbonate and activated carbon were kept constant at 0.7 and 0.07 g/100 ml, respectively. According to the experimental design, contact times ranging from 30 to 240 min were applied. After data collection, optimization tests were performed. The pH and acidity of the optimum treatment, the stock solution of tartaric acid (0.6% m/w), and the control sample were investigated. Also, precipitates of chemical adsorbents inside the samples were separated by filter paper and subjected to structural testing by FT‐IR.

### 2.4 pH

In order to determine the pH of the samples, the electrode of the AZ 86502 pH meter was put in contact with the samples before the pH values were recorded (Bruijn, Venegas, Martinez, & Borques, 2003).

### 2.5 Acidity

This test was performed using the potentiometric method. In brief, 50 ml of distilled water was mixed with 20 g of grape juice. Then, 0.1 N sodium hydroxide solution was added drop‐wise to bring the pH of the juice to 8.1; the volume of sodium hydroxide consumed was recorded, and the acidity of the samples was obtained by the following equation:

(2) $$A=\frac{V\times 0.0075\times 100}{m}$$

where *A* is the total acidity in terms of tartaric acid in grams per 100 ml; *V* is the volume of sodium hydroxide consumed (0.1 normal per ml), and m is the sample weight in grams (Hashim, Zubairi, Wan Mustapha, & Masket, 2018).

### 2.6 Deacidification rate

The rate of acid uptake by sorbents from grape juice samples was calculated using the following equation:

(3) $$A=\frac{C_{i}-C_{f}}{C_{i}}\times 100$$

where *C_i_* is the initial acidity, *C_f_* is the final acidity, and *A* is the acidity uptake percentage (Ghasempour, Alizadeh‐Khaledabad, Vardast, & Bari, 2016).

### 2.7 Turbidity

The Hach 2100AN Laboratory Turbidimeter was used to determine the turbidity of the samples after filtering (Bruijn et al., 2003).

### 2.8 Transmittance

In order to determine the transparency of the samples after filtering, a US‐based UV2100 spectrophotometer was employed at a wavelength of 625 nm. Distilled water was used as the blank (Bruijn et al., 2003).

### 2.9 Reducing sugars

The content of reducing sugars was estimated using the dinitrosalicyclic acid (DNS) method. The DNS method is a colorimetric technique that consists of a redox reaction between 3,5‐dinitrosalicyclic acid and the reducing sugars present in a sample. The reducing power of these sugars comes from their carbonyl group (C=O), which can be oxidized to the carboxyl group by mild oxidizing agents, while the DNS (yellow) is reduced to 3‐amino‐5‐nitrosalicylic acid (red‐brown) that can be quantified by spectrophotometry at 540 nm. The intensity of the color is proportional to the concentration of sugars. The reaction is carried out in an alkaline medium (Abdelmalek, Sila, Haddar, Bougatef, & Ayadi, 2017).

### 2.10 Polyphenol content

In order to measure the total polyphenol content, the Folin–Ciocalteu reagent method was used. In this experiment, the absorbance of the samples was measured at 765 nm. The phenolic compounds of the samples were calculated using the standard gallic acid curve. The amount of total phenolic compounds was expressed in milligrams of gallic acid equivalents per gram of dry sample. In this test, 200 µl of Folin reagent (10 times diluted), 1 ml of saturated sodium carbonate, and 1 ml of grape juice were mixed and stirred for all treatments before being incubated in the dark for half an hour. The absorbance of the samples was then evaluated. It should be noted that the blank sample employed contained the same amount of Folin reagent and sodium carbonate but 1 ml of distilled water instead of grape juice (Liu et al., 2019).

### 2.11 Formalin index

The first step of the procedure employed to evaluate formalin index included the addition of 25 ml of juice to a beaker. While stirring was performed, the juice was titrated drop‐wise to pH = 8.1 with sodium hydroxide. Then, 10 ml of neutral formaldehyde solution was added before the mixture was stirred for 1 min. Next, it was titrated with 0.1 N sodium hydroxide to pH = 8.1. The volume of sodium hydroxide consumed was recorded, and the formalin index was obtained using the following equation (Garcia, Barros, Fidalgo, & Ilharco, 2007).

(4) $$F=\frac{V\times N\times 10\times 100}{V_{0}}$$

where *F* denotes the formalin index, *V* is the volume of sodium hydroxide consumed (0.1 normal per ml), *N* is the normality of sodium hydroxide, and *V*_0_ is the sample volume in ml.

### 2.12 Brix

This test was performed using the JK‐ARM refractometer. The device was first set to zero with distilled water. Then, droplets of grape juice (20°C) were placed on a refractometer prism graded according to sucrose. The results were expressed in grams per gram of the samples (Liu et al., 2019).

### 2.13 Optimization

After analysis of the physicochemical properties, optimization was performed on the 16 samples related to the Box–Behnken experimental design (Table 1) using the response surface statistical method with the Design‐Expert 7 software. This optimization was done with the goal of achieving minimal acidity and turbidity rate but maximal acid adsorption rate, sample transmittance, reducing sugars content, and phenolic compounds content. After optimization, pH, acidity, and FT‐IR tests were performed on the optimum sample. Also, the stock solution of tartaric acid with acid concentration of 0.6 g/100 ml was prepared based on the total acidity of grape juice, and the adsorbents were added to the stock solution at optimum amounts. The pH, acidity, and FT‐IR tests were also performed on the filtrate of adsorbents separated from the stock solution as well those separated from the grape juice samples.

### 2.14 FT‐IR spectroscopy

FT‐IR spectroscopy of the chemical adsorbents was performed before and after contact with grape juice (Spectrum Two, Perkin Elmer). The chemical adsorbents were separated and filtered using filter paper before being placed in an oven set at 70°C. After drying the adsorbents, the optimum treatment of grape juice, the adsorbents of the stock solution, and the chemical adsorbents before contact were analyzed with FT‐IR. FT‐IR spectroscopy was run at a wavenumber ranging from 400 to 4,000 cm^−1^ with a resolution of 0.5 cm^−1^ (Ghasempour, Alizadeh‐Khaledabad, Vardast, & Bari, 2018).

## 3 RESULTS AND DISCUSSION

### 3.1 pH

According to the primary studies, the pH of the blank sample of grape juice was 3.84 before contact with any chemical adsorbent, improving to 6.51 after contact with calcium carbonate, nano‐silica, and activated carbon adsorbents; here, calcium carbonate was the most effective adsorbent. The findings of the second part of study showed that adding nano‐silica and alumina to samples across different contact times (30–240 min) had no significant effect on the pH of the examined grape juice. The pH remained at 4.5 in all treatments, which may be related to the presence of a constant amount of calcium carbonate within all treatments or to the existence of buffer substances such as proteins in the grape juice. Our findings are in accordance with those of the study of Kaiser (2012) in that the increase in pH was in line with the increase of calcium carbonate adsorbent content.

### 3.2 Acidity and acid adsorption

**FIGURE 1:**
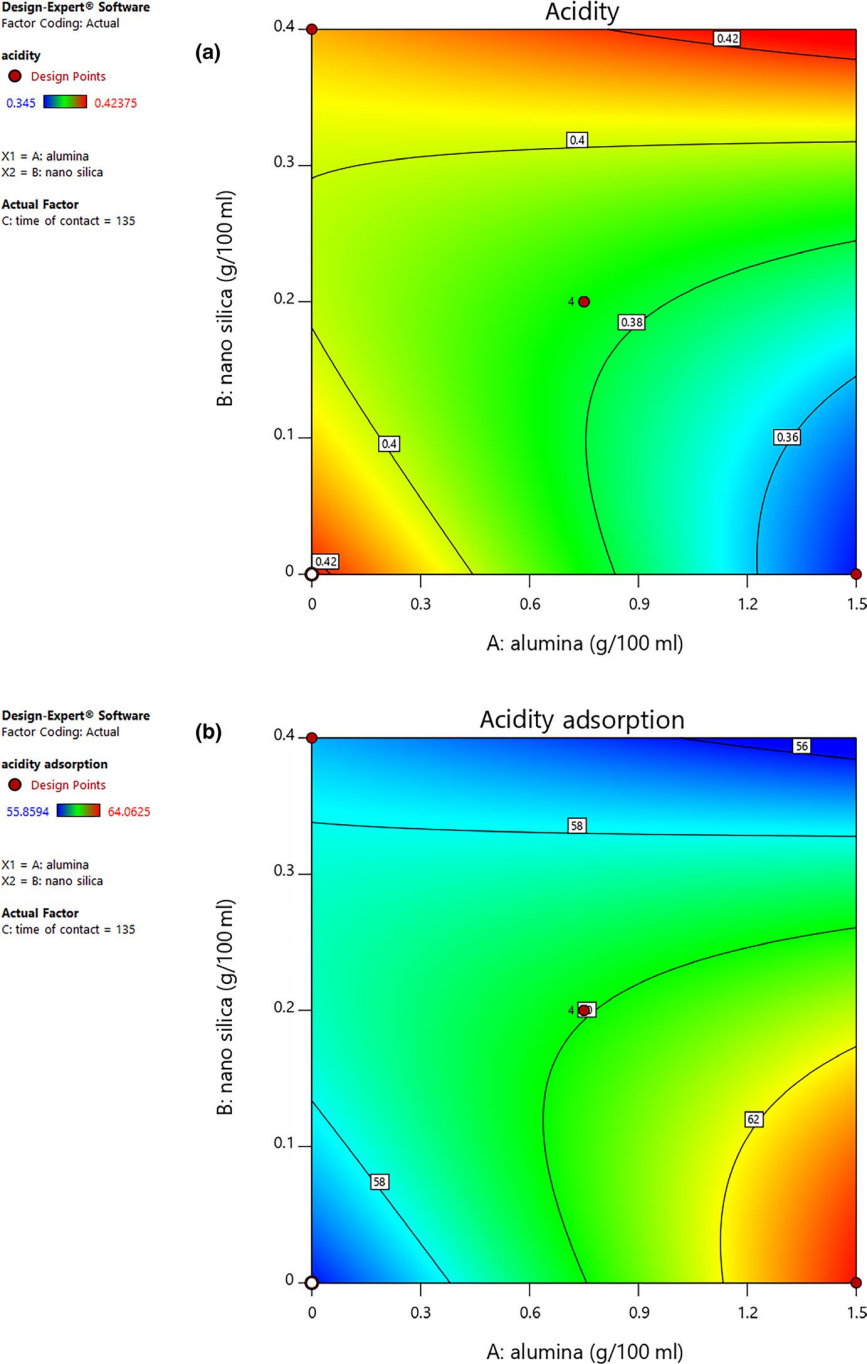
Contour plot curve of interaction effect of nano‐silica and alumina contents on acidity (a) and acidity adsorption (b)

In total, the acidity of the grapes was related to approximately 0.3% of grape juice content, mostly being due to tartaric acid (about 90%), with small amounts of malic and citric acid also being present. In the primary experiments, this amount decreased to 0.03% in the presence of a mixture of calcium carbonate, nano‐silica, alumina, and activated carbon adsorbents at levels of 0.74, 0.14, 0.02, and 0.1 g per 100 ml, respectively. In other words, the acidity was reduced by 10%. Figure 1a demonstrates the synergistic influence of nano‐silica and alumina on grape juice acidity. The findings prove that the grape juice acidity was reduced by increasing the contents of both nano‐silica and alumina. In fact, by increasing the nano‐silica level from 0.2 to 0.3 g per 100 ml, the acidity was gradually reduced by this adsorbent. The maximum amount of acid reduction was observed by adding 0.2 g of nano‐silica and 1.5 g of alumina; the interaction effect of nano‐silica and alumina on grape juice acidity reduction was highly significant (*p* < .05). However, the contact time had no significant effect on acidity (*p* > .05).

The amount of acid adsorbed by the adsorbents was measured through the acidity index of the samples, which was obtained using Equation 3; the total acid (mostly comprised of tartaric acid) adsorbed using nano‐silica and alumina adsorbents was thus determined. Figure 1b shows the effect of nano‐silica and alumina on grape juice acidity adsorption. The results show that the grape juice acidity adsorption increased with rises in both nano‐silica and alumina contents. It should be noticed that the slope related to alumina shows considerable differences compared to the nano‐silica adsorbent in acidity reduction. In addition, by increasing the nano‐silica from 0.2 to 0.3 g per 100 ml, acid adsorption was gradually increased by this adsorbent. In a research conducted by Uslu and Inci (2009), tartaric acid adsorption by alumina from stock solution was studied with the aim of separating tartaric acid from wastewaters of wineries. In their research, temperature was considered at three levels of 298, 310, and 325 K; the maximum percentage of tartaric acid adsorption (22%) took place at 298 K.

In our experiment, the addition of different amounts of nano‐silica and alumina adsorbents caused reductions in acidity level of grape juice samples mixed with calcium carbonate and activated carbon while increasing the level of acidity adsorbed by the adsorbents (*p* < .05). According to the efficiency of alumina and nano‐silica adsorbents in isolation or in combination, it seems that the mechanism of acid adsorption is the formation of hydrogen bonds between hydrogen and the tartaric and malic acid molecules by means of oxygen atoms available in the chemical compounds of alumina (Al_2_O_3_) and silica (SiO_2_). Our results are in accordance with the research of Uslu and Inci (2009) considering the issues that have already been mentioned. Our findings are also in agreement with those of Wisniewska, Urban, Grządka, Zarko, and Gunko (2014), who reported similar surface adsorption of polyacrylic acid by mixture of nano‐silica and alumina by values of 4% and 96%, respectively.

### 3.3 Transmittance

Transmittance refers to transferred energy through foods, which is related to the amount of light diffusion by matter. Transmittance testing was used to show the amount of clarity (i.e., lack of impurity) though the scattering of radiated light across grape juice samples after the addition nano‐silica and alumina adsorbents. The findings indicate that the effect of nano‐silica and alumina contents on the transparency factor of grape juice was significant (*p* < .05), but the contact time variable had no significant effect (*p* > .05). With an increase in the amount of alumina, the transparency of the tested samples decreased. Aluminum oxide is chemically neutral and usually has low interactivity. For this reason, alumina may remain in the fruit juice and reduce the transparency of the samples. In contrast, the transparency of the tested samples increased with a rise in nano‐silica content. Due to its negative surface charges, silicate can adsorb positively charged proteins and eliminate protein colloids from solutions (Belitz, Grosch, & Schieberle, 2009; Filho & Carmo, 2004).

### 3.4 Turbidity

**FIGURE 2:**
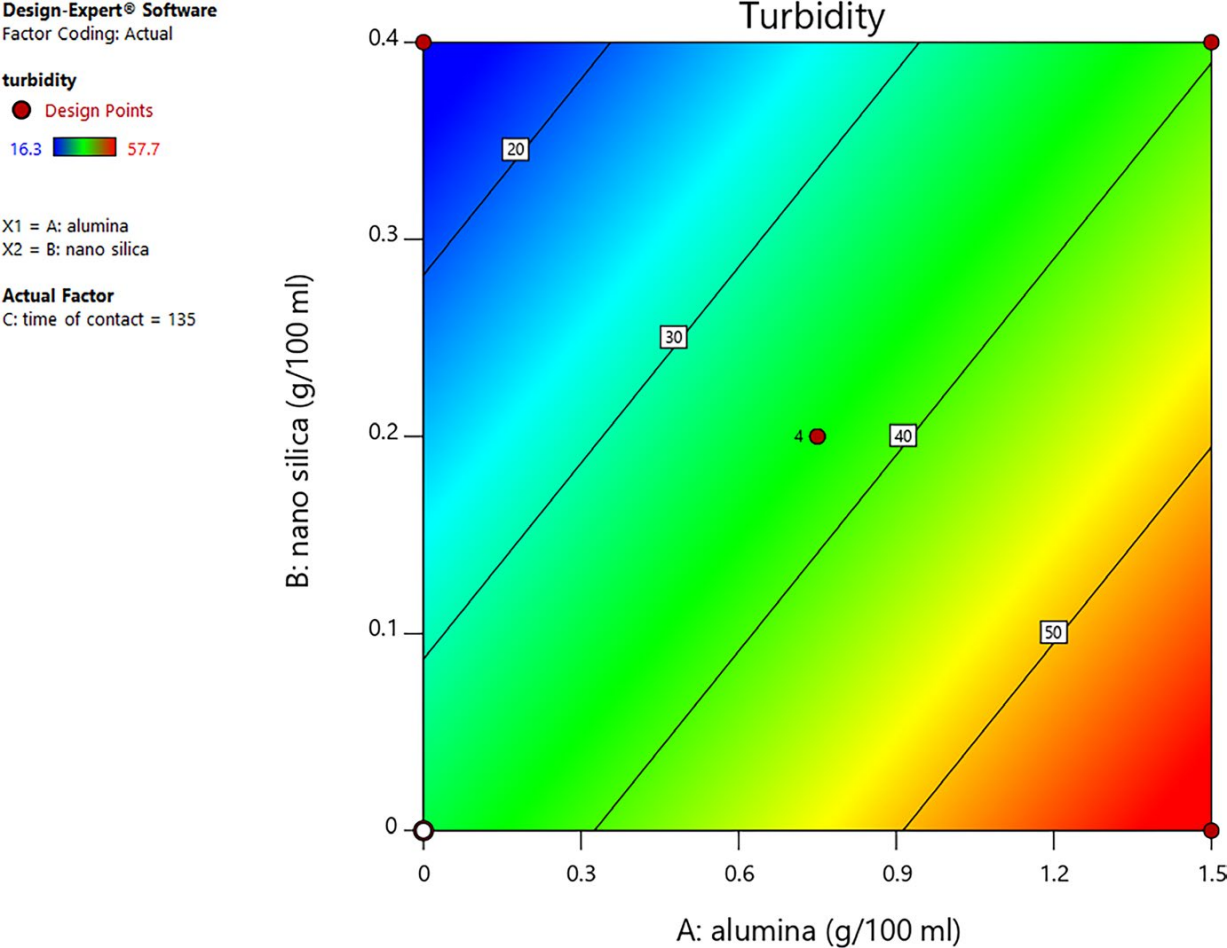
Contour plot curve of interaction effect of nano‐silica and alumina contents on turbidity

Turbidity is an indicator that shows the amount of light transmission through fluids and liquids. This indicator expresses the turbidity level of food samples in nephelometric turbidity units (NTU). The reason for turbidity is the existence of some suspended particles or insoluble solids such as food colloids, proteins, and phenols. Figure 2 demonstrates the interaction effect of nano‐silica and alumina contents on grape juice turbidity. The results show that the grape juice turbidity considerably decreased with increasing nano‐silica and decreasing alumina contents. Therefore, the effect of nano‐silica and alumina on the turbidity factor of grape juice samples was highly significant (*p* < .05), but contact time had no significant effect (*p* > .05). According to the findings of Aljohani et al. (2018), it seems that the reason for the reduction in grape juice turbidity with increased nano‐silica content is the adsorption of proteins and phenolic compounds by silica (Aljohani et al., 2018).

### 3.5 Reducing sugars

The obtained results clarified that the effects of alumina adsorbent and contact time variables as well as their interaction on the content of reducing sugars were significant in grape juice samples (*p* < .05). The results also showed that with a simultaneous rise in the time period and amount of alumina, the content of reducing sugars first decreased but then increased. Alumina, in the amount of 0.3 to 0.9 g per 100 ml, caused the adsorption and reduction of reducing sugars in grape juice samples. Since all monosaccharaides are reducing sugars, and considering the report of Singh and Mohan (2004) about the weak acid behavior of monosaccharaides inside water solutions, it seems that in the present study, the reason for the fall in the content of reducing sugars in the presence of alumina was the adsorption of reducing sugars by alumina via hydrogen bonds. The later rise in reducing sugars may be attributed to the release of the adsorbed sugars during longer contact times. The findings confirm that the usage of large amounts of alumina adsorbent and a contact time of 240 min have beneficial and desired effects on the content of reducing sugars of samples (Singh & Mohan, 2004).

### 3.6 Total phenolic compounds (TPC)

Nowadays, it is known that phenolic compounds have protective effects on the antioxidative capacity of biological systems due to reducing hydrogen peroxidation, activating antioxidant enzymes, reducing α tocopherol radicals, and inhibiting oxidases. Besides, phenolics are suitable antimicrobial, antiviral, and anticancer compounds. Several studies have reported many polyphenols found in grapes such as flavan‐3‐ols, quercetin, and anthocyanins (Pasini, Riciputi, Fiorini, & Caboni, 2019). However, removal of phenolics and proteins during grape juice production is essential, otherwise they cause the hazing of grape products, which is maintained by their interactions (Kulcan, Öziyci, Tetik, & Karhan, 2015). In this experiment, the changes occurring in the TPC amount of the samples was investigated. The findings reveal that the minimum amount of TPC could be obtained by addition of 0.1% alumina and 0.1% nano‐silica; this reduction occurred at maximum amount of nano‐silica and alumina and at 1.5% alumina and 0.4% nano‐silica, showing the synergistic effect of those sorbents. The results indicate that during the contact time, the phenolic compounds content first fell before increasing. In general, the results prove that 30‐min contact time, 0.4 g per 100 ml nano‐silica content and 1.4 g per 100 ml alumina content had a minimum but desired effect on phenolic compounds' adsorption. Besides, in the grape juice samples, the interaction effect between the nano‐silica and alumina adsorbents on total phenol content was significantly affected by contact time (*p* < .05).

### 3.7 Formalin index

**FIGURE 3:**
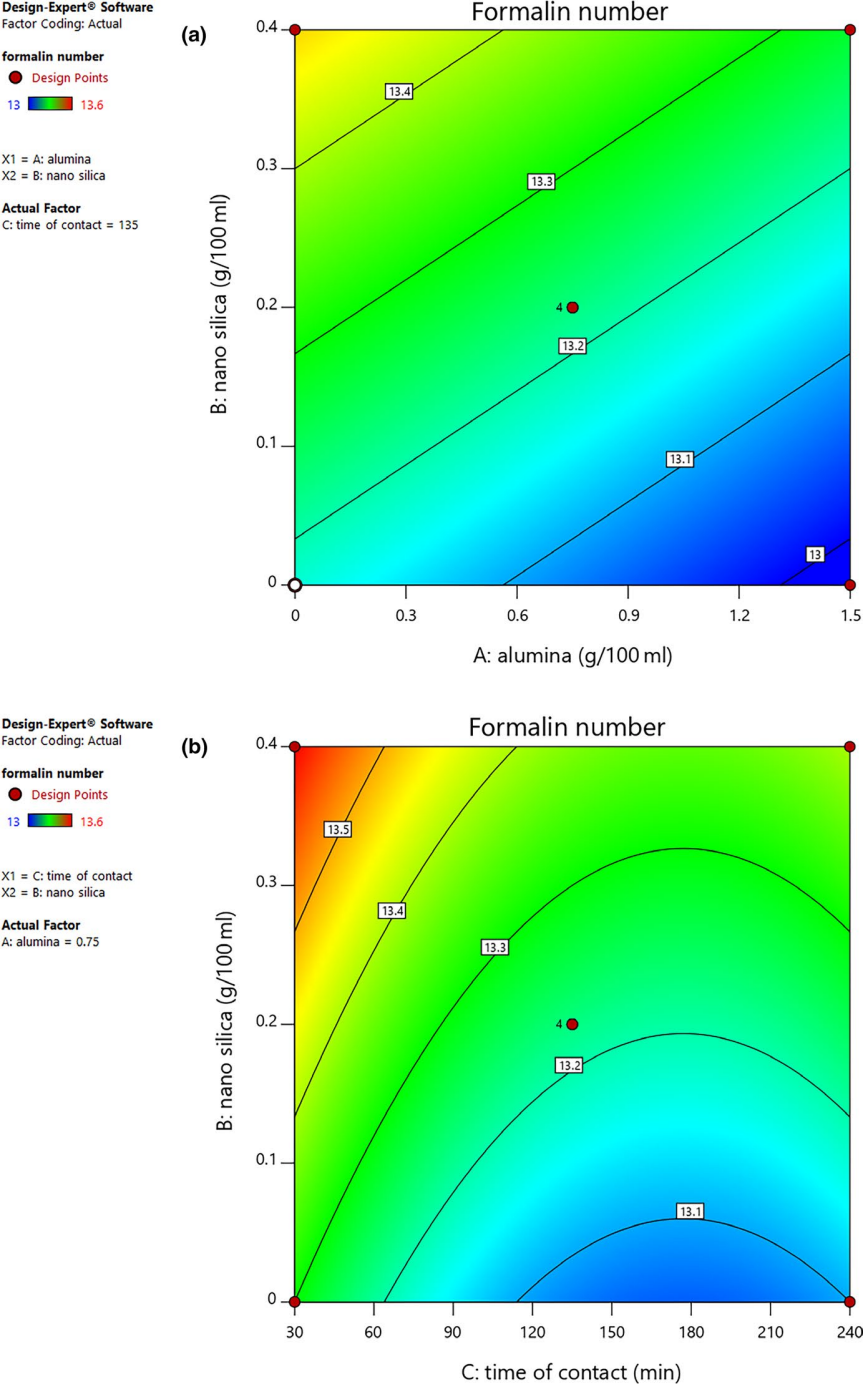
Contour plot curve of interaction effects of nano‐silica and alumina (a) as well as nano‐silica and contact time (b) on Formalin index

Formalin index is known as the indicator of amino acid presence in fruit juice products. Figure 3a shows that the effect of the two variables of alumina and nano‐silica contents on the index factor of formalin for grape juice. The obtained results indicate that the formalin index of the samples decreased with an increase in alumina amount and a decrease in nano‐silica amount. Furthermore, Figure 3b depicts the fall in formalin index with decreased nano‐silica content and a rise in formalin index with increased contact time; the highest formalin index value was observed at highest value of nano‐silica content. These findings demonstrate that the maximal value of nano‐silica content (0.4 g per 100 ml) and minimal contact time (up to 1 hr) are desirable. In addition, according to ANOVA results, the influences of the two variables of nano‐silica and alumina as well as contact time on factor of formalin for grape juice samples were significant (*p* < .05). Our results are in line with those of Garcia et al. (2007) who expressed that the reason for the adsorption and decline in L‐alanine amino acid by alumina in water solutions is the formation of hydrogen bonds between the alanine amino acid and the Al‐OH_2_^+^ groups.

### 3.8 Brix

Brix indicates the amount of soluble solids of samples. A change in the level of Brix of samples is possible if any additional soluble fractions are incorporated into the grape juice. Therefore, this test was done to guarantee the state of change of Brix by adding used adsorbents. The results show that none of the variables under investigation had any significant effects on Brix (*p* > .05). In fact, the lack of changes in the soluble solids content after going through the neutralization stages and acid adsorption from grape juice samples was desirable (*p* > .05). The findings of this research are similar to those of Liu et al. (2019), who observed no change in the Brix of apple juice samples after contact with chemical adsorbents.

### 3.9 Optimization

Considering all results, the optimization was done in two steps. Firstly, the primary test was performed based on the desired objectives. Furthermore, based on the acidity of nontreated grape juice, a solution of tartaric acid was obtained at the same amount of acidity (0.6 g per 100 ml). In the second step, by selecting optimum amounts of adsorbents, the effect of the optimum treatment on pH, acidity, and FT‐IR spectroscopy was studied on grape juice and tartaric acid solution. The aim of this procedure was to determine the optimum mixture of applied adsorbents in order to decrease tartaric acid adsorption and allow a minimal level of adsorption of nutritional compounds such as phenolic compounds. Table 2 shows the conditions of the optimized treatment.

**TABLE 2:** Optimized treatment conditions with the goal of achieving minimal acidity and turbidity rate but maximal acid adsorption rate, sample transmittance, reducing sugars content, and phenolic compounds content in grape juice deacidification with adsorbents

Alumina (g/100 ml)	Nano‐silica (g/100 ml)	CaCO_3_ (g/100 ml)	Activated Carbon (g/100 ml)	Contact time (min)	pH	Acidity (%)	Acidity uptake (%)	Transmittance (%)	Turbidity (NTU)	Reducing sugar (mg/ml)	Polyphenol content (mg/100 ml)	Formalin index	Brix	Desirability
1.27	0.21	0.7	0.07	239.9	4.3	0.37	60.81	86.31	45.37	17.91	106.63	13.18	24.8	0.71

### 3.10 FT‐IR spectroscopy

**FIGURE 4:**
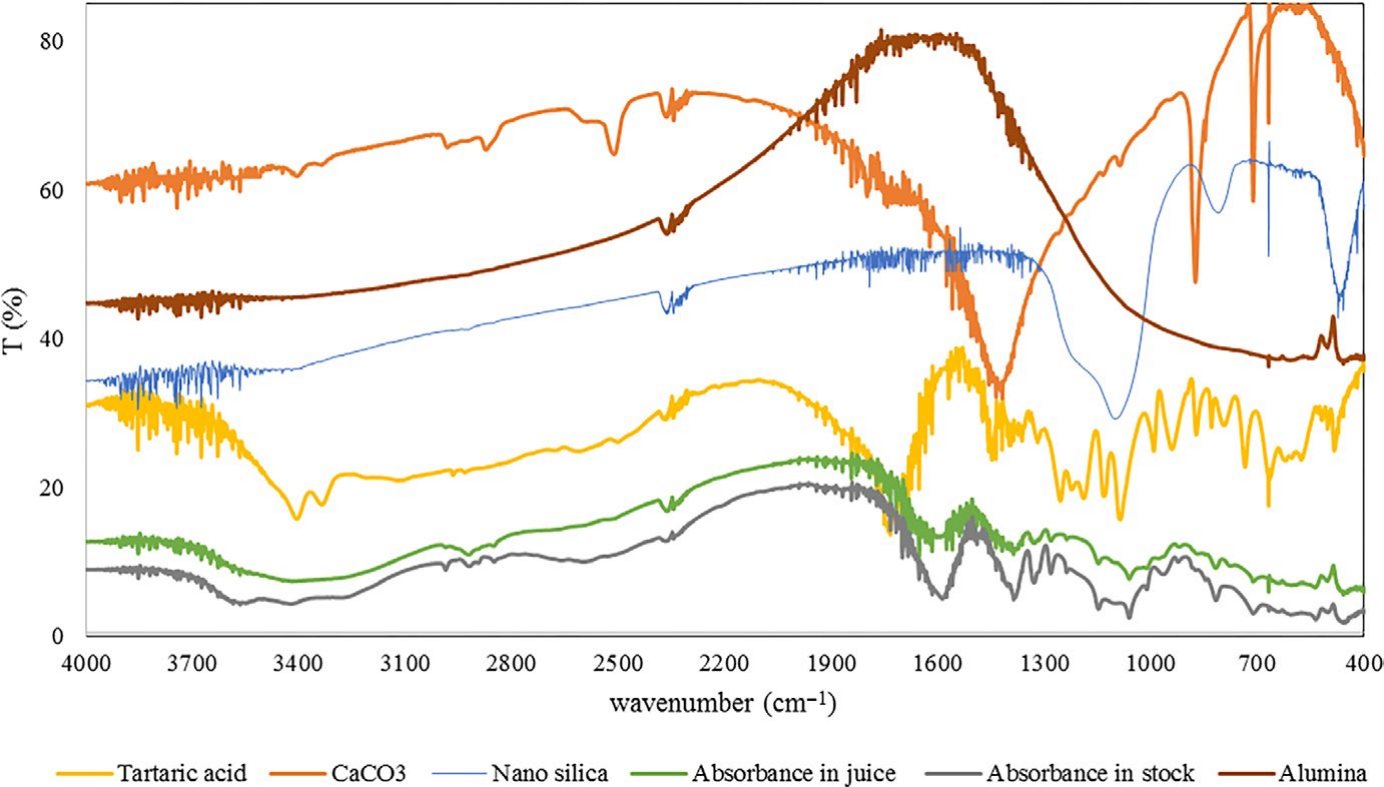
FT‐IR spectra related to: tartaric acid, nano‐silica, calcium carbonate, and alumina before contact; mixture of optimum adsorbents after contact with grape juice; and spectrum of mixture of optimum adsorbents after contact with stock solution

FT‐IR spectroscopy is applied as an appropriate approach to determine the functional groups and or chemical bonds of a material. Figure 4 demonstrates the FT‐IR spectra related to tartaric acid, nano‐silica, calcium carbonate, alumina, and activated carbon before contact, as well as those of the mixture of optimum adsorbents after contact with grape juice and of the mixture of optimum adsorbents after contact with stock solution.

Sharp peaks at 2,508 and 3,407 cm^−1^ in calcium carbonate and at 3,552 cm^−1^ in tartaric acid became broader in the spectra related to grape juice and stock solution, with all four adsorbents being shifted; this denotes the formation of O‐H bonds and adsorption of tartaric acid. The peaks related to C‐C‐H bonds (2,947 cm^−1^) and C‐C triple‐bonds (2,358 cm^−1^) were available and constant across all spectra. The wide peaks formed at a wavelength of 1,596 cm^−1^ indicate C=C and C=O bonds, which were formed in the adsorbents after contact with grape juice and stock solution of tartaric acid. These peaks were sharper in the spectra related to all adsorbents ahead of contact. Furthermore, in the postcontact spectra, the wide peaks formed at around 711 cm^−1^ show C‐O and C‐C bonds after contact with grape juice and stock solution of tartaric acid; this peak was sharper in the latter. Considering the results of FT‐IR test and the formed bonds, adsorption of tartaric acid was accomplished. According to the efficiency of alumina and nano‐silica adsorbents in isolation or in combination, it seems that the reason for formation of new hydrogen bonds in the IR spectrum was related to the mixture of optimum adsorbents and the adsorption of tartaric acid by alumina (Al_2_O_3_) and silica (SiO_2_). The obtained results for tartaric acid are in accordance with the research of Ghasempour et al. (2018).

### 3.11 pH and acidity

The pH of the stock solution and grape juice samples was evaluated before and after contact with the optimum mixture of adsorbents. The pH of the stock solution was initially 2.5, which increased to 3.39 after contact with optimal adsorbents. Also, the pH of grape juice was initially 3.2, which increased to 4.6 after contact with optimal adsorbents. According to the obtained results, the amount of tartaric acid in the stock solution was 0.675 g per 100 ml, which decreased to 0.154 g per 100 ml after contact with optimal adsorbents. Additionally, the total acidity of the control grape juice sample attributable to tartaric acid was 0.615 g per 100 ml, which decreased to 0.176 g per 100 ml after contact with optimal adsorbents.

## 4 CONCLUSION

The objective of this research was to separate tartaric acid from grape juice by usage of a novel adsorbent mixture of nano‐silica (nanoparticles of SiO_2_), calcium carbonate (CaCO_3_), alumina (Al_2_O_3_), and activated carbon in order to produce grape syrup, which was characterized during contact time. The applied adsorbents were studied in order to obtain low‐cost and safe adsorbents with high efficacy for the replacement of traditional methods with a glance of mass industrial production. The calcium carbonate (0.7%) and activated carbon (0.07%) contents were kept constant across all treatments, while nano‐silica and alumina adsorbents were varied in content and examined in the contact time of 30–240 min. The evaluated parameters were pH, total acidity, reducing sugars, transmittance, total phenol compounds, turbidity, formalin index, and Brix. Also, after optimization of the pH value, the acidity, and FT‐IR spectra were determined for the optimal adsorbents after contact with grape juice and tartaric acid solutions. According to the results of this study, the effects of nano‐silica and alumina on Brix and pH were not significant (*p* > .05). In addition, an increase in the amount of nano‐silica adsorbent had a significant effect on the increase of transmittance and decrease of turbidity in the samples in contact with chemical adsorbents. The results show that an optimal adsorption rate of 60% was achieved for the adsorption of total grape juice acidity through 240 min of contact with 0.21 g/100 ml of nano‐silica, 1.27 g/100 ml of alumina, 0.7 g/100 ml of calcium carbonate, and 0.07 g/100 ml of activated carbon. Hence, the usage of this optimal adsorbent mixture could be introduced in the processing of industrial grape syrup products in order to enhance the efficacy of clarification.

## CONFLICT OF INTEREST

The authors declare that they do not have any conflict of interest.

## ETHICAL STATEMENT

This study does not involve any human or animal testing.

## INFORMED CONSENT

Written informed consent was obtained from all study participants.
